# Re-evaluation of the role of Indian germplasm as center of melon diversification based on genotyping-by-sequencing analysis

**DOI:** 10.1186/s12864-019-5784-0

**Published:** 2019-06-03

**Authors:** Maria José Gonzalo, Aurora Díaz, Narinder P. S. Dhillon, Umesh K. Reddy, Belén Picó, Antonio J. Monforte

**Affiliations:** 1 Instituto de Biología Molecular y Celular de Plantas, Universitat Politècnica de València-Consejo Superior de Investigaciones Científicas, Valencia, Spain; 2 Unidad de Hortofruticultura, Instituto Agroalimentario de Aragón (IA2) (CITA-Universidad de Zaragoza), Avenida de Montañana 930, 50059 Zaragoza, Spain; 3 World Vegetable Center East and Southeast Asia/Oceania, Kasetsart University, Kamphaeng Saen, Nakhon Pathom, 73140 Thailand; 4 Department of Biology, Gus R. Douglass Institute, West Virginia State University, Institute, WV 25112-1000 USA; 5 Instituto de Conservación y Mejora de la Agrodiversidad Valenciana, Universitat Politècnica de València (COMAV-UPV), Valencia, Spain

**Keywords:** *Cucumis melo*, SNP, Haplotype, Genetic structure, Diversity, Linkage disequilibrium

## Abstract

**Background:**

The importance of Indian germplasm as origin and primary center of diversity of cultivated melon is widely accepted. Genetic diversity of several collections from Indian has been studied previously, although an integrated analysis of these collections in a global diversity perspective has not been possible. In this study, a sample of Indian collections together with a selection of world-wide cultivars to analyze the genetic diversity structure based on Genotype by Sequence data.

**Results:**

A set of 6158 informative Single Nucleotide Polymorphism (SNP) in 175 melon accessions was generated. Melon germplasm could be classified into six major groups, in concordance with horticultural groups. Indian group was in the center of the diversity plot, with the highest genetic diversity. No strict genetic differentiation between wild and cultivated accessions was appreciated in this group. Genomic regions likely involved in the process of diversification were also found. Interestingly, some SNPs differentiating inodorus and cantalupensis groups are linked to Quantitiative Trait Loci involved in ripening behavior (a major characteristic that differentiate those groups). Linkage disequilibrium was found to be low (17 kb), with more rapid decay in euchromatic (8 kb) than heterochromatic (30 kb) regions, demonstrating that recombination events do occur within heterochromatn, although at lower frequency than in euchromatin. Concomitantly, haplotype blocks were relatively small (59 kb). Some of those haplotype blocks were found fixed in different melon groups, being therefore candidate regions that are involved in the diversification of melon cultivars.

**Conclusions:**

The results support the hypothesis that India is the primary center of diversity of melon, Occidental and Far-East cultivars have been developed by divergent selection. Indian germplasm is genetically distinct from African germplasm, supporting independent domestication events. The current set of traditional Indian accessions may be considered as a population rather than a standard collection of fixed landraces with high intercrossing between cultivated and wild melons.

**Electronic supplementary material:**

The online version of this article (10.1186/s12864-019-5784-0) contains supplementary material, which is available to authorized users.

## Background

Melon (*Cucumis melo* L.) is one of the most important fruit crop species belonging to the Cucurbitaceae family. Nowadays, melon is cultivated worldwide and extensive phenotypic variability is found among modern and traditional cultivars, landraces, feral and wild plants. Several intraspecific classifications have been proposed based on morphological traits [1, 2]. In the last proposed classification, 19 horticultural groups were defined: agrestis, kachri, chito, tibish, acidulus, momordica, conomon, makuwa, chinensis, flexuosus, chate, dudaim, chandalak, indicus, ameri, cassaba, ibericus, inodorus, and cantalupensis [3]. Concomitantly, molecular markers have been used to study the distribution of the genetic diversity in the species [4–6]. In general, molecular diversity analyses cluster cultivars in groups that fit the morphological ones, which indicates that the Mediterranean/Near East groups (ameri, cassaba, chandalak, ameri, ibericus, inodorus, cantalupensis) are closely related and quite differentiated from Far-East groups (conomon, makuwa, chinensis).

*C. melo* is native to Asia [7], although the history of domestication and diversification is not clear yet [3]. Wild melons are found in both Africa and Asia (India), what could be explained by the migration of wild melons from Asia to Africa. African and Indian cultivars/landraces are clustered with wild melons from their respective regions [6], what support at least two domestication events. Domestication of melons in Asia would have led to the diversification of most melon horticultural groups that spread rapidly westwards to the Mediterranean Basin in ancient times, as supported by *C. melo* seeds found which date from pre-dynastic Egypt [8–10]. Domestication in Africa would have had a marginal impact, as only tibish cultivars would have been generated from this event [11]. Recently, Endl et al. [12] also supported the two domestication events and the role of Asian domestication in the origin of most current melon cultivars. Therefore, Central Asia can be considered the best candidate region for the major domestication and diversification events.

Indian melon genetic diversity has been studied thoroughly with Single Sequence Repeat (SSR) markers in four collections from different regions of India: landraces North India (IC collection, [13]), East India (AM collection, [14]), South India (SM collection, [15]), and wild genotypes from North India (WM collection, [16]), together with reference accessions (from makuwa to inodorus). Most of the Indian collections showed a lateral position with the reference accession in the genetic diversity plot, except for the IC collection from North India [13]. These results suggested that the IC collection could represent a center of melon diversity, i. e., crop diversified in a divergent way from India to Mediterranean and Far East regions from North India. The rest of the collections could represent genetic diversity that would have remained mostly in India. In order to confirm this hypothesis, it is necessary to take a broader view of genetic variability by including these Indian collections together with a larger array of melon accessions. The integration of IC collection matrix data [13] with the rest of Indian collections was not possible as the SSR markers used for the experiments were different, hampering the study of the genetic relationship between the IC and AM, SM and WM collections. Other published works used different marker systems which makes it impossible for data integration.

Genotyping-by-Sequencing (GBS) approaches [17] have become popular as an efficient and affordable method to obtain a large number of marker genotypes for different genetic studies [18]. The most common approach consists of genome complexity reduction by restriction enzyme digestion and adapter ligation before sequencing. We decided to re-analyze a sample of those Indian collections with reference cultivars by GBS. Recently, Nimmakayala et al. [19] analyzed by GBS a collection of 120 melon accessions with a good representation of occidental horticultural groups (cantalupensis, inodorus,… etc.) and some Far-East accessions (makuwa and conomon horticultural groups) although with a low representation of Indian accessions. This collection perfectly complements the previously reported Indian collection. GBS library construction can produce different representation of the genome, although part of the represented genome may be common between independent experiments, making the integration possible. In the current report, we obtained GBS data from a sample of the IC-, AM-, SM- and WM- collections as well as 50 additional reference accessions and merged these with Nimmakayala et al. [19] data. Thus, a genotypic data matrix with sufficient Single Nucleotide Polymorphism (SNP) information and representing major melon horticultural groups was obtained to test the hypothesis on the origin of melon diversification in Central Asia. Furthermore, the generated data was also used to gain some insights into the evolution of the melon genome through cultivar diversification and identification of candidate genomic regions that could be involved in the process.

## Results

### SNP discovery by genotyping-by-sequencing

The GBS analysis of the 78 new IBMCP melon accessions (Additional file 1: Table S1) resulted in 146,367 SNPs with at least three reads of support (Additional file 2; Table S2). After filtering for Minor Allele Frequency (MAF) higher than 0.05 and frequency of missing data lower than 0.05, the retained number of SNPs was 8215, similar to the 7609 SNPs retained after filtering the WVSU SNP data set [19], which demonstrates that the quality of both data sets was similar. All the SNPs were integrated before filtering from both datasets in order to retain more SNPs., A total of 8212 common SNPs were found among data sets. The merged matrix was filtered for MAF > 0.05 and presence of SNP data in at least 100 accessions, remaining 6169 SNPs. Finally, 11 SNPs showed a heterozygosity larger than 0.7, likely an artifact due to sequencing errors or duplicated sequences, and they were also removed. Finally, 6158 SNPs were retained, with a mean of 513 SNP/chromosome, ranging from 657 SNP in chromosome 1 to 337 SNP in chromosome 10. The SNP density was 12.8 SNP/Mb.

### Genetic structure and diversity

**Fig. 1:**
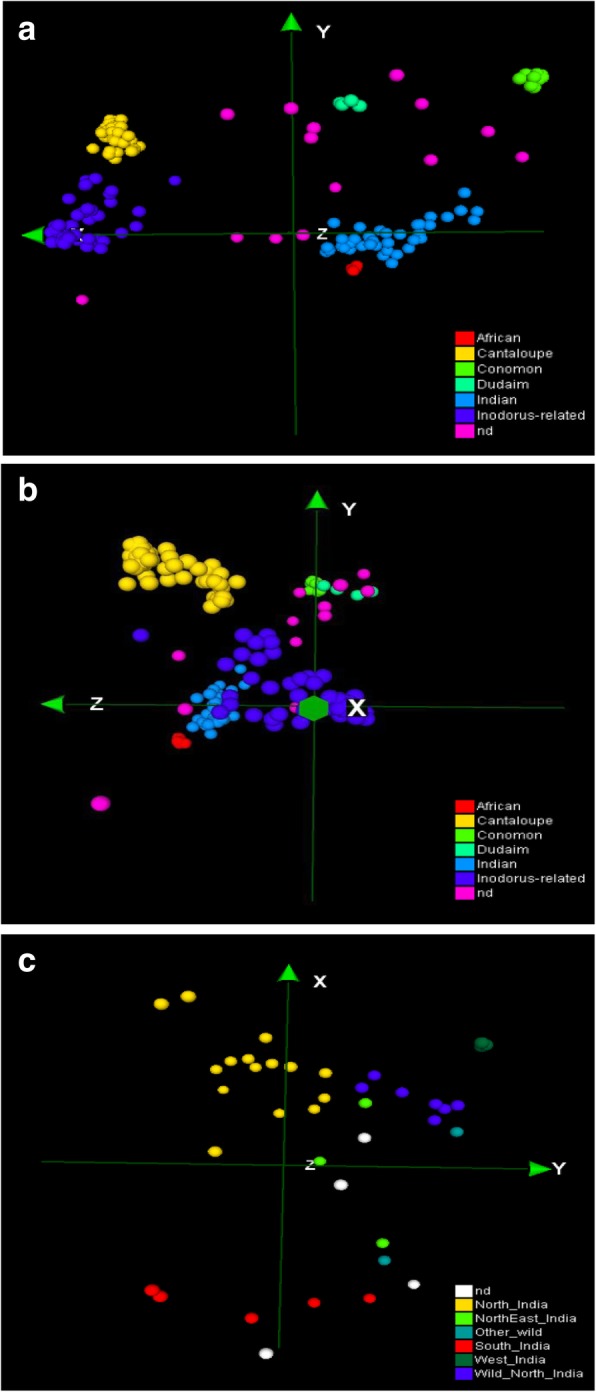
Multidimensional scaling (MDS) of different groups of accessions based on GBS SNPs. (**a**) and (**b**) depict the XY and XZ axis, respectively, for the whole germplasm collections, which define the groups highlighted with colors as indicated in the legend (nd indicates accessions that were not assigned to any group). (**c**) MDS focused only on Indian germplasm, indicating the subgroups according to the legend

Multidimensional scaling (MDS) analysis was carried out to investigate the genetic structure of the current melon germplasm. A preliminary analysis was performed to verify the absence of bias due to GBS data merging. We found that accessions from the same horticultural group (cantalupensis, conomon, dudaim and inodorus) from the two data sets plotted in the same MDS space region, indicating that the possible bias for merging was negligible (Additional file 3: Figure S1). Furthermore, the novelty of the Indian germplasm included in the current report is also evident in this figure. Subsequent analysis were performed after pruning the genotype matrix for linkage disequilibrium, resulting in a final matrix with 4661 SNPs. The three first MDS axis explained 85% of the variance, 75, 7 and 3% the axis X, Y and Z, respectively. The MDS plot (Fig. 1a) depicted a diversity distribution through the X-axis that is concomitant with the geographic origin of the accessions: Mediterranean/Near East accessions on the left, Indian in the center towards the right and conomom (China and Far East) on the right. Integrating the X and Y MDS axis, six groups could be defined: cantaloupe (including cantalupensis cultivars), inodorus-related (flexuosus, inodurus, ameri cultivars and landraces), dudaim (dudaim landraces), Indian (acidulus, chito, snapmelons and wild Indian accessions) and conomon (makuwa and conomon cultivars and landraces) and accessions of African origin. The Z-axis also differentiated African accessions and cantaloupes from the rest (Fig. 1b) and cantaloupe. STRUCTURE analysis showed that K = 5 had the highest peak based on Delta K distribution, supporting the groups defined by MDS (Additional file 4: Figure S2), except of dudaim that appeared as a mixture population.

Cantaloupe and inodorus-related groups were located in the left part of the MDS plot, consistent with their classification in the *C. melo* subsp. *melo*. These two groups were distinguished only in the Y-axis. STRUCTURE separated both groups at K = 4 and K = 5 (Additional file 4: Figure S2). In general, the pre-defined classification of cultivar type fitted very well with the MDS and STRUCTURE groups, except for a few cantalupensis and reticulatus types which were grouped in the inodorus-related MDS group, what could be explained as a misclassification (for example Honeydew and Ananas melons usually are classified as inodorus) as most of them are traditional cultivars with properties that may not fit exactly with the modern commercial types.

Most of the pre-defined dudaim accessions were included in a MDS group (named afterwards as dudaim group), except three of them that were ungrouped and ‘Jenny Lind’ which grouped with the cantaloupe group. This group, despite of being classified initially as *C. melo* subsp. *melo* (Additional file 1: Table S1), was located in the center of the plot with other *C. melo* subsp. *agrestis* accessions/landraces (Fig. 1a). STRUCTURE analysis did not allow a clear assignation of dudaim landraces in any subspecies. For K = 2, dudaim group seemed to be more related to Indian, conomon and African groups (all of them belonging to subsp. *agrestis*), however, for K > 3 the group seems to be a mixture of several populations (Additional file 4: Figure S2.). A larger sample of these landraces would be necessary to classify them firmly in any of the subspecies. Conomon and makuwa cultivars and landraces plotted together on the right part of the MDS plot, showing the highest distance from the cantaloupe and inodorus-related groups (Fig. 1a). STRUCTURE analysis also classified these groups as a single defined population for K > 3.

Indian and African groups were located in the middle of the plot, although slightly separated, and clearly differentiated from the other groups (Fig. 1b, Additional file 4: Figure S2.). STRUCTURE analysis (K = 5) also supported a separation of African and Indian accessions (Additional file 4: Figure S2.). Two African accessions (CO148 and TGR1551) gathered together with the Indian group what likely reflects migration from India. Wild melons from Africa and India were included in different STRUCTURE populations that are related with their geographical origin, with their respective cultivated accessions.

A detailed MDS analysis within the Indian group was performed (Fig. 1c). The Indian populations IC (North India), SM (North East India), AM (South India), and WM (Wild North India) were grouped according their geographic origin. Wild North Indian group (WM) was slightly separated from the cultivated North Indian group (IC), although the degree of separation was much lower than the separation of IC group from the other cultivated Indian groups (AM, SM), and one accession from North-East_India cluster with Wild_Norhth_India.

### Genetic diversity

Two levels of classification were defined for the AMOVA analysis: two subspecies (*C. melo* subsp. *melo* and subsp. *agrestis*) and six groups (African, cantaloupe, conomon, dudaim, Indian and inodorus-related), based on MDS results. The highest percentage of variation, 58.49%, was due to the diversity within groups (Table 1a). Variation between subspecies (*C. melo agrestis* vs *melo*) was 22.29%, and the variation among groups within subspecies was 19.23% of the total variance. Variation among groups according to F_st_ was 41% of the total genetic variation. The AMOVA done only with the groups also reflected that around 60% of the genetic diversity was within the defined groups (Table 1b).

**Table 1:** Distribution of genetic variability based on MANOVA analysis. (A) including the subspecies and MDS groups levels and (B) only the MDS groups

Source of variation	Sum of squares	Variance components	Percentage variation	*P*
A				
Among subspecies	34,000.11	321.74	21.97	< 0.00001
Among groups within subspecies	26,061.30	264.03	18.03	< 0.00001
Within groups	136,176.52	878.56	60.00	
TOTAL	196,237.93	1464.33		
B				
Among groups	60,061.41	462.33	34.48	< 0.00001
Within groups	136,176.52	878.56	65.52	
TOTAL	196,237.93	1340.88		

**Fig. 2:**
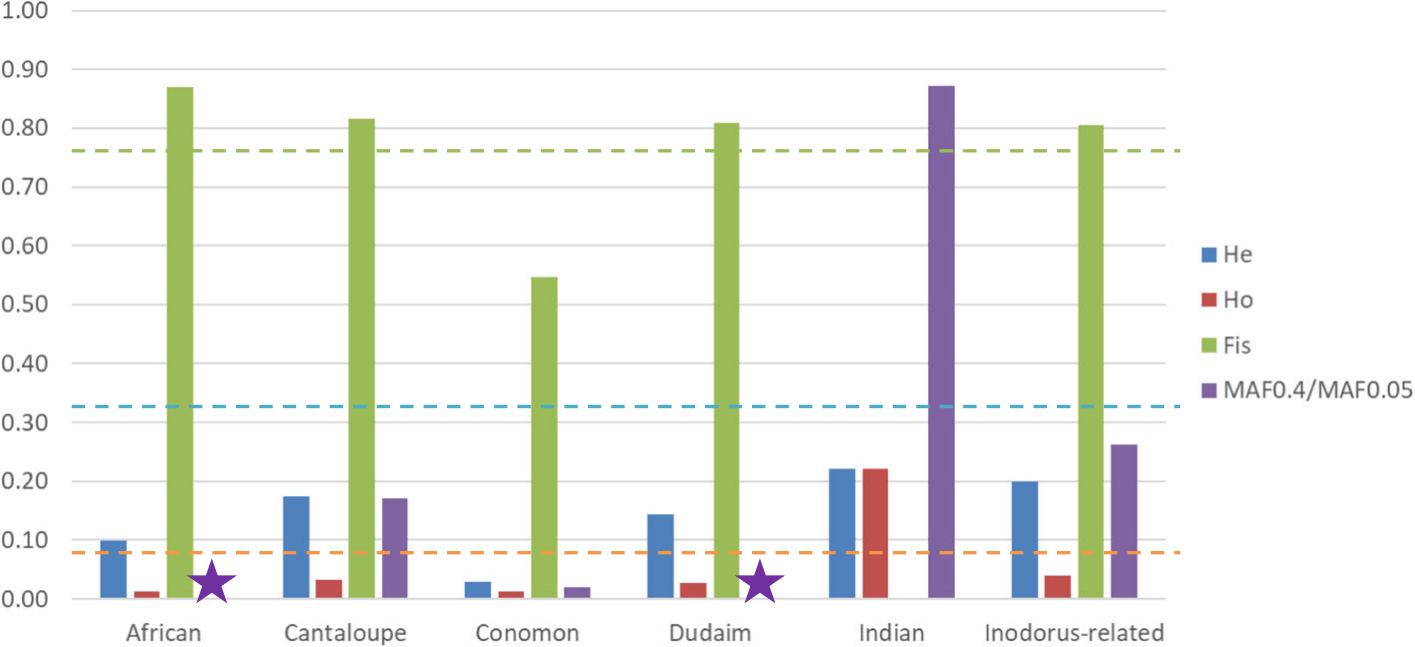
Observed (H_o_) and expected (H_e_) heterozygosity fixation index (F_is_) and the ratio (MAF0.4/MAF0.05) between the number of SNPs with minor allele frequency higher than 0.4 (MAF0.4, i. e., highly variable) and number of SNPs with MAF lower than 0.05 (MAF0.05 i. e. very low variability) for the MDS-defined groups. This ratio was not calculated for Dudaim and African groups due to the low representation from these groups (indicated with a star). The overall mean is indicated with a dashed line for each estimate

The average H_e_ was 0.32, with the highest value for Indian, followed by inodorus-related and cantaloupe groups (Fig. 2). The distribution of H_e_ through the genome was quite variable among them. In Inodorus-related and cantaloupe groups, genomic regions with low or no diversity (H_e_ ≃ 0) were detected to be scattered through the genome in all chromosomes (Additional file 5: Figure S3). In the case of Indian group, this low diversity was less frequent than in the previous groups. Most of the genome showed low H_e_ within the conomon group (data not shown).

On the other hand, H_o_ was very low (average H_o_ = 0.08) in most groups, except for the Indian group where it was equal to H_e_ (H_e_ = H_o_ = 0.22) (Fig. 2). Concomitantly, the fixation index (F_is_) distribution showed a reverse pattern.

The ratio between the frequency of high polymorphic SNPs (MAF > 0.4) and low polymorphic SNPs (MAF < 0.05) was also calculated (MAF0.4/MAF0.05) to obtain further details on the distribution of genetic diversity within the groups. MAF0.4/MAF0.05 ratios were low in all the groups (even nearly 0 for the conomon group), except for the Indian group where MAF0.4/MAF0.05 was high. The frequency of SNPs with different MAF also varied drastically among the groups. For the whole collection, SNP frequency at different MAF showed low variability, ranging from 0.15 to 0.22 (Additional file 6: Figure S4.). On the other hand, SNPs with MAF > 0.1 were majority in the conomon group (92%), 49 and 57% in inodorus-related and cantaloupe groups, respectively, whereas it was only 28% in Indian group. In the first three groups, the frequency of SNPs with MAF > 0.1 decreased drastically, however in the Indian group the decrease of SNP frequency was observed only from MAF > 0.3, and was very less pronounced than in the previous groups.

**Fig. 3:**
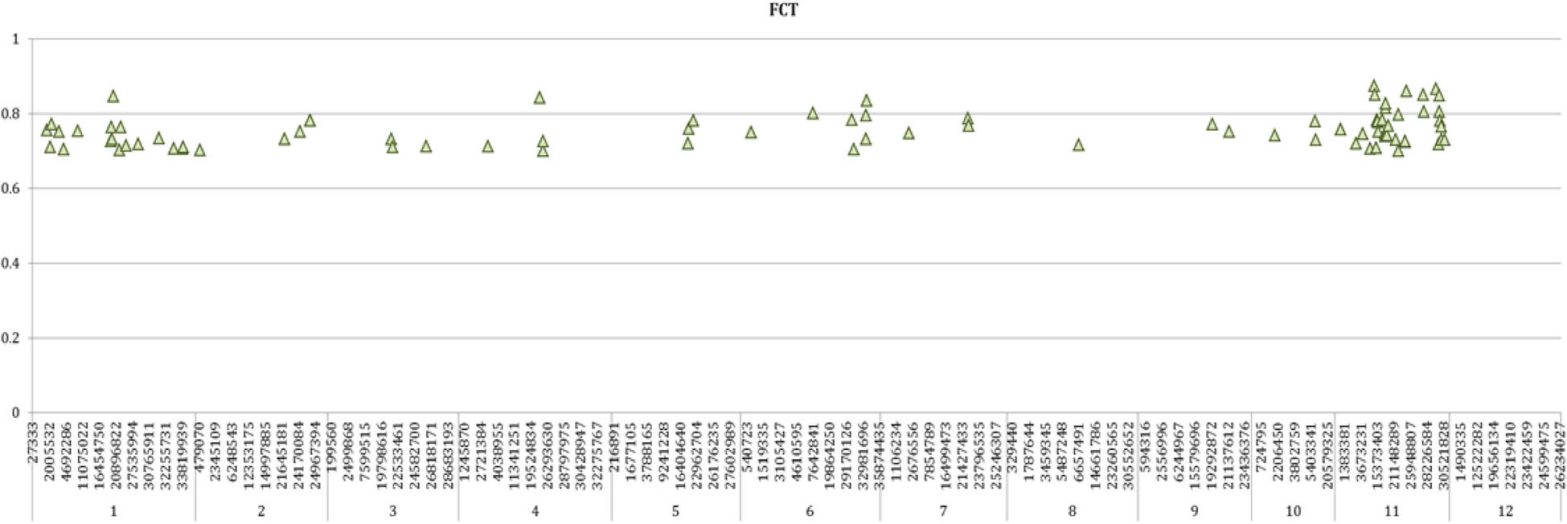
Genomic position on the 12 melon chromosomes of SNPs with Fct (genetic differentiation between subspecies *C. melo* subsp. *agrestis* and *C. melo* subsp. *melo*) > 0.70

Finally, the Wright’s F statistics were analyzed to find candidate loci under selection during the diversification to subspecies and groups. The coefficient of genetic differentiation among subspecies (F_ct_) explained 22.29% of the genetic variation between melons from the two subspecies, *C. melo* subsp. *melo* and *C. melo* subsp. *agrestis*. There was a considerable variation of F_ct_ across the genome, nevertheless, 39 SNPs showed high levels of F_ct_ (> 0.70) with loci located throughout all the chromosomes except for chromosome12 (Fig. 3). Chromosomes 1, 6 and especially 11 showed higher numbers of SNPs with high F_CT_.

**Fig. 4:**
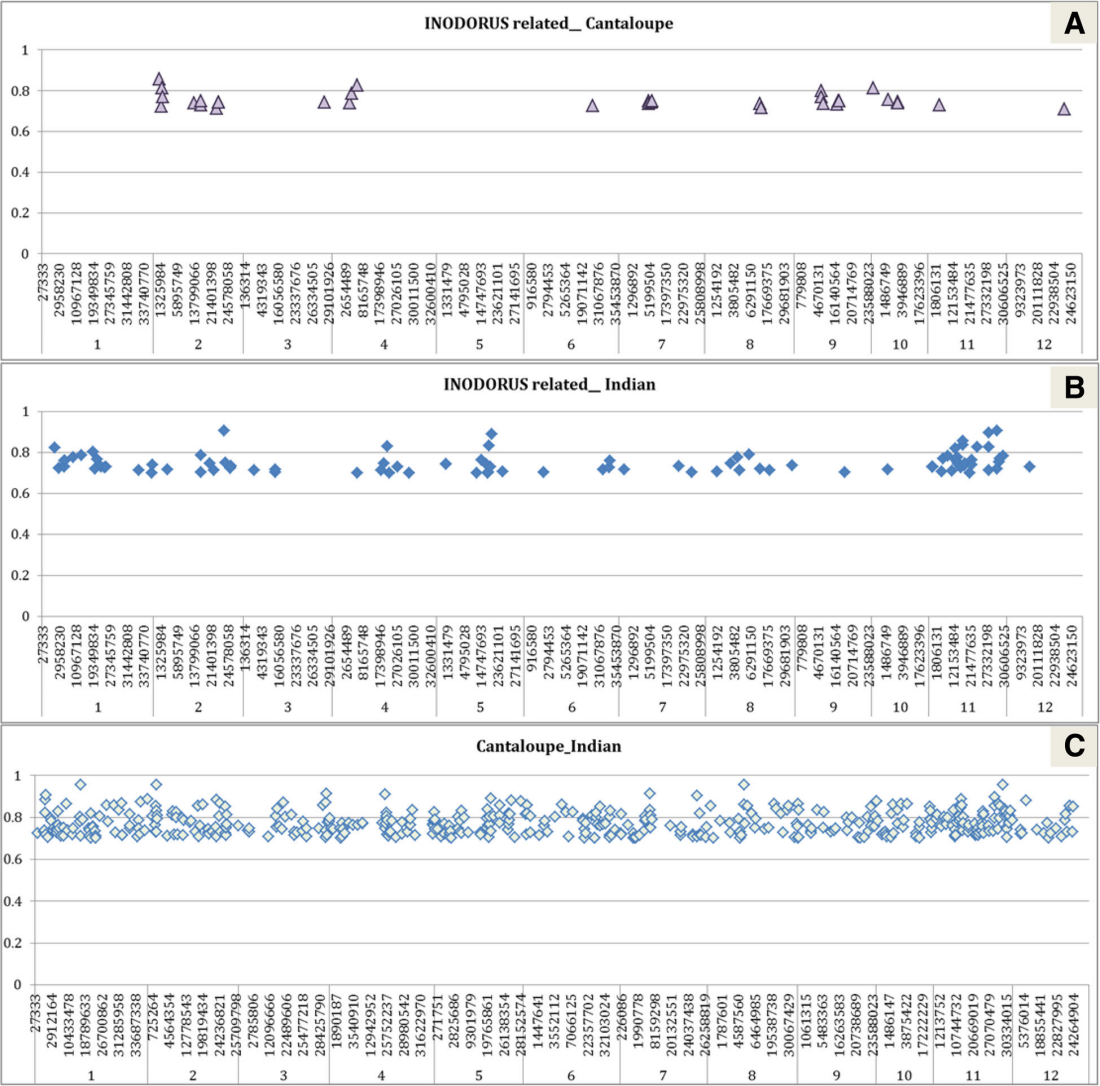
Genomic position on the 12 melon chromosomes of SNPs with Fst (genetic differentiation between melon groups> 0.70. **a** inodorus_related vs cantaloupe, (**b**) inodorus_related vs Indian and (**c**) cantaloupe vs Indian groups

The pair-wise F_st_ statistic, that is related to genetic differentiation among groups, was calculated between groups with sufficient representation (inodorus-related, cantaloupe and Indian, Fig. 4 Additional file 7: Table S3.). In the pairwise comparison between inodorus-related and cantaloupe groups 306 SNPs with F_st_ > 0.70 were found to be distributed through chromosomes 2, 3, 4, 6, 7, 8, 9 10, 11 and 12 (Fig. 4a). Between inodorus-related and Indian groups (Fig. 4b), 93 SNPs showed F_st_ > 0.70. SNPs with the highest F_st_ were found in chromosomes 1, 2, 4, 5, 6, 8, and 11. The comparison between the cantaloupe and Indian groups displayed 471 SNPs with F_st_ > 0.70, distributed in all the chromosomes (Fig. 4c). Nevertheless, SNPs with the highest F_st_ were coincident with the previous pairwise comparison.

#### Linkage disequilibrium and haplotype blocks

**Fig. 5:**
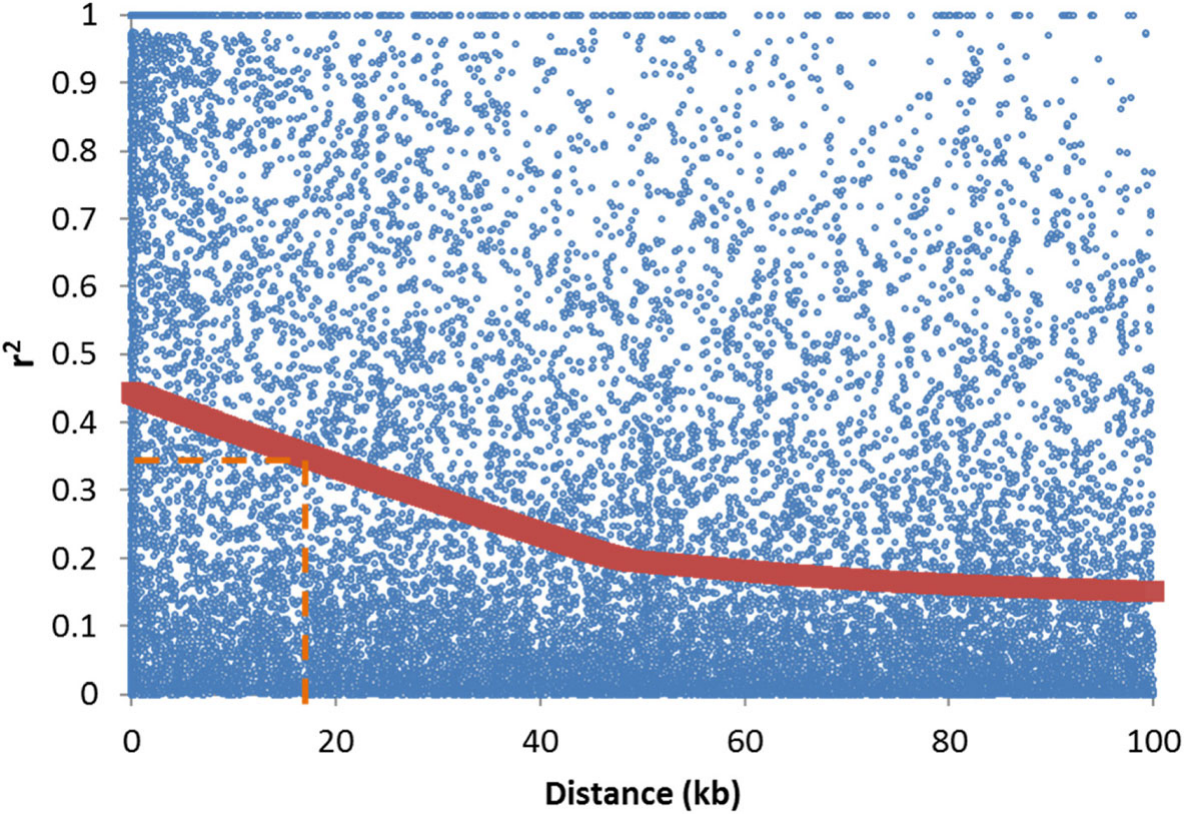
Linkage disequilibrium (r^2^) versus physical distance (kb) in the whole germplasm collection. Dashed line indicates the false discovery rate at *p* < 0.05 based on the 95th percentile of the r2 distribution of unlinked SNPs. The curve was fitted by second-degree LOESS

LD was analyzed initially in the whole germplasm collection (Fig. 5). LD was very variable among different genomic windows. LOESS regression was applied for pairwise distances lower than 100 kb, which showed that, in general, LD was low (r^2^ < 0.5) even at very close distances (< 1 kb) and it decreased steadily up to 40 kb approximately. The 95th percentile of the r^2^ among unlinked SNP was 0.35, corresponding to a physical distance of 17 kb (Fig. 5). LD was further analyzed separately for euchromatic and heterochromatic genome regions. The LD decay in the euchromatin was sharper, with a rapid decay up to 12 Kb, and, in this case, the 95th percentile of r^2^ among unlinked SNP corresponded to 8 Kb. On the other hand, there was no clear LD decay with distance, except for very close distances, in the heterochromatin regions, being nearly constant around *r*^2^ = 0.35 (Additional file 8: Figure S5A). LOESS window was increased to 200 kb. LD decay with the distance was clearer in this new settings, reaching the threshold value r^2^ < 0.35 at 30 kb when the LD was also calculated separately for the inodorus-related and cantaloupe groups defined by the MDS. In these cases, LD was more important, with r^2^ > 0.7 at very short distances, and the r^2^ threshold (*r*^2^ = 0.2) reached 49 and 97 Kb for inodurus-related and cantaloupe groups, respectively (Additional file 8: Figure S5B).

A total of 1128 haplotype blocks were identified by SNPanalyzer. The mean haplotype size was 59 kb, although most of them were small: only 9 (0.8%) were larger than 1 Mb, 40% smaller than 1 kb, being the median 4.5 kb (Additional file 9: Table S4). A larger number of haplotypes were detected in euchromatin when compared with heterochromatin (847 vs 281, Additional file 10: Figure S6), but smaller in size (mean haplotype size 31 kb) than in the heterochromatin (mean haplotype size 147 kb). In fact, eight out of a total of nine haplotypes larger than 1 Mb were located in heterochromatin regions.

The haplotype block frequency within and between inodorus-related, cantaloupe, conomon and Indian groups and the two subspecies was studied more thoroughly. A total of 17 haplotypes were found to be fixed in one of the groups/subspecies but segregating among other groups. The size of the haplotypes was variable (from 4.7 to 875 kb) and most of them were located in euchromatic regions (Table 2) Three haplotypes were found to be fixed in inodorus-related, five in cantaloupes and nine were characteristic of *C. melo* subsp. *melo* cultivars, and therefore are candidate regions for involvement in the diversification of melon cultivars.

**Table 2:** Chromosomal location (CM3.51 genome coordinates indicating euchromatine (EU) or heterochromatine (HET) regions) of haplotypes fixed either in cantaloups or inodorus groups compared with the rest of germplasm groups The letters A, B, C indicates different haplotypes. A > B indicates that first haplotype is nearly fixed. A + B indicates that both haplotypes are present (not necesarily at the similar frequency)

Genomic position						Haplotypes				
chromosome	scaffold	start	end	HET/EU	size	INODURUS	CANTA	CONOMON	Indian	Group
1	CM3.5.1_scaffold00017	19,507,561	19,515,103	HET	7542	A	A + B	B	B + other no A	Inodorus
1	CM3.5.1_scaffold00026	30,564,508	30,569,227	EU	4719	A	A	B	A + B	melo
3	CM3.5.1_scaffold00014	25,454,999	25,616,076	EU	161,077	A > B	A	B	A + B	Canta
4	CM3.5.1_scaffold01596	2,674,636	2,677,147	EU	2511	A	A	B	A + B	melo
4	CM3.5.1_scaffold01596	3,112,230	3,162,514	EU	50,284	A > B	A > B	B	A + B	melo
5	CM3.5.1_scaffold00022	2,797,288	2,825,686	EU	28,398	A + B	B > A	B	B > A others	melo
5	CM3.5.1_scaffold00003	26,134,651	26,181,558	EU	46,907	A	A	B	A + B	melo
6	CM3.5.1_scaffold00078	29,170,126	29,280,943	HET	110,817	A	A	B	Recombinant	melo
6	CM3.5.1_scaffold00021	31,589,587	31,627,509	EU	37,922	A > B	A > B	B	No A No B	melo
7	CM3.5.1_scaffold00035	15,470,961	15,599,403	HET	445,734	A	A	B	A > B	melo
9	CM3.5.1_scaffold00051	1,143,401	1,167,001	EU	23,600	A	A	B	A + B	melo
9	CM3.5.1_scaffold00005	16,258,884	16,885,818	EU	626,934	A	A + B	C	A + B + C	Inodorus
10	CM3.5.1_scaffold00016	68,527	94,131	EU	25,604	A + B	A	A	A + B	Canta
10	CM3.5.1_scaffold00016	3,870,253	3,946,988	EU	76,735	A + B	A	B	A + B	Canta
11	CM3.5.1_scaffold00047	28,501,830	28,830,113	EU	878,141	A + B	A	C	A + C	Canta
11	CM3.5.1_scaffold00052	29,283,061	29,379,971	EU	96,910	A + C	A	B	A + C	Canta
11	CM3.5.1_scaffold00052	30,012,076	30,219,034	EU	206,958	A	A	B	A + B	melo
12	CM3.5.1_scaffold00001	24,407,798	24,426,009	EU	18,211	A	A > B	B	A + B	Inodorus

## Discussion

### SNP discovery by genotyping by sequencing and merging

In the current report, data from two independent GBS experiments were merged. The final number of common SNPs after filtering (6169 SNPs) was significantly lower than the SNPs detected in each individual experiment and in previous melon GBS reports [19–21]. A proportion of detected SNPs is expected to be experiment-specific, as the reduction of genome complexity based on restriction enzyme digestion and subsequent ligation and PCR-amplification for sequencing library construction may generate different genome representation between independent experiments. Nevertheless, the number of common SNPs was sufficient to perform population genetics analysis. One important concern was the possible bias of the independent experiments in classifying the accessions. Fortunately, a preliminary classification showed that cultivars belonging to known horticultural groups clustered together in the MDS plot, which confirmed that there was no bias or it was negligible. Therefore, merging GBS data from independent experiments seems to be an appropriate strategy at least for population genetic analyses.

### Genetic diversity

Previous SNP-based genetic diversity analysis of melon germplasm focused mainly on occidental germplasm (mainly inodorus, cantalupensis, ameri and flexuosus) with a relatively small representation of Far-East (conomon, makuwa, chinensis) and, especially, Indian germplasm [6, 19–23]. The general picture of all these works is similar: Occidental germplasm appears to be closely related, although with the major groups, inodorus and cantaloupe were clearly distinct at genetic level. Far East conomon is at the other extreme of the genetic variation space, while assorted accessions with Near East to Eastern origin are dispersed in the central space. Focusing only on the accessions that belong to the same horticultural groups in the current and previous reports, the general picture is the same, what confirms the reliability of the classification with the current data set.

The main novelty of the current report is the inclusion of Indian germplasm that had not been studied previously in a global array of melon accessions. MDS analysis showed that the Indian germplasm is located in the center of the MDS space as a compact but wide group. African germplasm is closely related, but STRUCTURE analysis supports its differentiation from Indian germplasm. The fact that cultivated and wild accessions are grouped by origin (African and India) indicates at least two domestication events occurring independently in the two continents, as proposed previously [11, 12]. Indian germplasm showed the highest level of genetic diversity, which also supports this region being the primary center of diversity of Euro-Asian melon cultivars. Occidental and Oriental cultivars were derived from India through a divergent selection process. Similar divergent diversification was found previously in cucumber [24]. Interestingly, genetic diversity in inodurus-related and cantaloupe groups remains relatively high, whereas in the conomon group it is very low. Bottleneck may have occurred during the diversification of conomon group cultivars, although this fact should be verified with a larger sample. On the other hand, the bottleneck that occurred during the diversification of inodorus-related and cantaloupe group cultivars seemed to be not so strong. However, the frequency of SNPs with high MAF have indeed reduced in inodorus-related and cantaloupe groups when compared with the Indian group. This result reflects a different distribution of the genetic variability in these groups. In the Indian group, the variability is shared among a high proportion of landraces, whereas in the rest of the groups it is fixed in a low number of landraces/cultivars. This fixation could be a consequence of farmer selection and development of homogeneous cultivars. The cultivars have been maintained by different farmers communities, have adapted to their different needs, and therefore, rare alleles are not eliminated by drift, but maintained in those selected cultivars.

Another interesting result is the difference in F_is_ among groups. F_is_ is high in all the groups except in the Indian group, in which it is nearly 0. High F_is_ has been reported often in inodorus, cantalupensis and conomon (including chinensis and makuwa) accessions [5, 6, 13, 25]. In accordance with it, low F_is_ has been recently reported in landraces collected directly in fields cultivated by traditional farmers, as snapmelons from North India [13], Xinjiang landraces in China [26] and snake melons from Israel and Palestine [27]. Several reasons may explain these differences: selfing during the seed multiplication in germplasm banks, a more intensive selection pressure of traditional farmers in order to keep fruit characteristics homogeneity in the traditional inodorus, cantalupensis and conomon cultivars avoiding intercrossing when compared with the other groups, and the different sex expression, most traditional cultivars from the last groups are andromonoecious, what would facilitate self-pollinated seeds compared with monoecious cultivars as the Indian and the traditional snake melons [3].

A relatively large number of SNPs with high F_st_ was found in three pairwise group comparisons (inodorus-related vs cantaloupe, inodorus-related vs Indian and cantaloupe vs Indian). The largest number of SNPs with a high F_st_ value was found in the cantaloupe vs Indian comparison. Interestingly, the SNPs with the highest F_st_ value (F_st_ > 0.75) between inodorus-related and Indian groups also showed a high F_st_ between cantaloupe and Indian groups (chromosomes 1, 2, 4, 5, 6, 8, 11). These are therefore good candidates to mark genomic regions that were fixed (either by selection or by drift) during the diversification from India to the Mediterranean Basin. It is remarkable that an important proportion of SNPs with a high F_st_ value were found on chromosome 11. Estimation of F_st_ among melon groups have been investigated previously in few reports [6, 28]. Given the different germplasm studied, especially the novel Indian germplasm included in the present work, comparisons among reports are not straight forward. F_st_ values for SNPs between cantaloupe and inodorus collections were also reported by Esteras et al. [6]. Four of their reported SNPs (Cmpsnp1067 on chromosome 2, fr14p22 on chromosome 6, Cmpsnp590 on chromosome 9, Cmpsnp1109 on chromosome 12) are located in the same genomic regions where SNPs were found with high F_st_ among the groups, which supports the hypothesis that those regions may be involved in the differentiation of the groups of cultivars. Moreover, one of these SNPs (fr14p22) is located in the *CmNAC-NOR* gene, a member of the NAC-domain TF family, that is the gene underlying the QTL for climacteric ripening *ETHQV6.3* [29, 30]. Fruits from i*nodorus* and *cantalupensis* varieties have different ripening behaviors: non-climacteric and climacteric, respectively. An SNP was also found on chromosome 3 (position 28,689,022) differentiating those groups located in the region of other climacteric QTL: *ETHQB3.5* [31]. These co-locations of differentiating SNPs among these groups and QTL that are involved in one of the major traits that differentiated the two groups validate the current strategy and the robustness of the other differentiating SNPs reported.

### Linkage disequilibrium and haplotype blocks

Previous reports on LD in melon have shown that LD decays rapidly within a few kb [6, 20, 23]. The threshold to declare significant LD was different in those reports due probably to the different germplasm used (threshold was higher in the two first works), but the general picture is similar. In the current report, significant LD extent for the whole collection was 18 kb. LD extent was dependent on the genomic region (9 kb for euchromatin, 40 kb for heterochromatin) and groups (50 kb for inodorus-related, 100 kb for cantaloupe). These results may explain the slight differences with the previous works. Nevertheless, most genes are located in euchromatic regions, where LD is much lower than the average genome-wide LD, which indicates that a very dense SNP panel would be necessary to ensure high linkage among SNPs for performing GWAS (Genome-Wide Association Studies).

LD in heterochromatin was higher, but still, it was low. Heterochromatic regions show no recombination in melon biparental populations [32], but the current results demonstrate that recombination exists in these regions. This result contrasts with that reported in other species as barley [33], potato [34] and tomato [35, 36] where large haplotype LD blocks are found in heterochromatic regions.

Concomitantly, haplotype blocks were generally small, with a large proportion of them located in heterochromatic regions, as found previously by [19]. Nevertheless, the number of haplotype blocks in centromeres was also low and large regions of centromeres did not show any block at all. However, most of the haplotypes that were fixed in varieties that belong to one subspecies or group (Table 2) were located in euchromatic regions, where LD is much less intense than in heterochromatin. This apparent contradiction may indicate that those regions are good candidates to include genes involved in melon diversification.

Thus, LD in the melon genome can be considered as relatively low. The extent of LD depends on the genome region under study due to the recombination suppression, for instance, in the centromeric regions. Anyway, the LD in the centromeric regions can also be considered low (r2 ≈ 0.3), indicating that recombination has occurred in those regions during the history of melon cultivation, even though recombinants usually are not found in the common mapping populations [26].

Given the low LD observed in the present study (the genotyped SNPs do not cover all the genomic regions), other regions carrying genes also involved in cultivar diversification may have been missed. Higher SNP density would be necessary to obtain a full picture of the dynamics of melon genome during the diversification, which, for example, could be obtained by resequencing.

### Indian germplasm in the context of melon diversity

Accessions representing several Indian collections that have been previously investigated separately have been studied to have a better picture of the genetic diversity distribution in these collections using GBS-based SNP markers. In the current report, those accessions have been integrated and contextualized using a truly representative sample of global melon germplasm. Indian germplasm appears as a group of related accessions, clearly separated from other international accessions, and it has a central position in the *C. melo* genetic variability space. Looking with more detail into the distribution of the genetic variability among Indian accessions, this group of accessions can be separated more clearly by geographical origin than cultivated/wild types, which indicates that probably there is no strict genetic differentiation between wild and cultivated melons in India. Indian germplasm is the group with the highest genetic diversity, indicated by high SNP variability, high proportion of SNPs with high MAF and low haplotype fixation. Its heterozygosity level was as expected under H-W equilibrium, whereas in the other groups it was very low, probably as a consequence of farmer selection to maintain cultivar homogeneity for desired traits. In fact, the low proportion of SNPs with high MAF in the other groups confirms that rare alleles are fixed in few cultivars/landraces, not shared among other cultivars, and maintained as consequence of farmer selection of specific cultivars. The situation in Indian group is radically different, the high proportion of SNPs with high MAF (in cultivated and wild melons) supports that the genetic variability is not fixed within landraces, but it is shared among them. Thus, the Indian germplasm represented here may not be a standard collection of landraces (as for inodurus-related and cantaloupe groups), but a population of plants with extensive intercrossing in the field among wild and cultivated melons that would maintain the high genetic diversity. An interesting question is why did not Indian traditional farmers caused gene fixation in landraces, whereas high values of the fixation index are relatively common in landraces from both extremes of the melon geographical distribution. One possible factor for this may be the sex determination of the female flower, being strictly female in Indian landraces (and therefore obligatory out-crossers) and hermaphrodite in the most of rest, which facilitates selfing. The co-existence of wild and cultivated melons in Indian fields would produce frequent intercrosses and making difficult the fixation of alleles in specific landraces.

## Conclusions

In summary, our results confirm the crucial importance of India as a center of melon diversity. Germplasm origination in India differs from African germplasm. Mediterranean and Far-East traditional cultivars would have developed from Indian germplasm by a divergent diversification. Melons cultivated by local Indian communities might be in the early steps of domestication with genetic exchange with wild melons as there is low evidence of genetic differentiation from the wild melons and the low degree of fixation. These results suggest that the germplasm may be considered genetically as a population of plants, rather than as a standard group of landraces.

## Methods

### Plant material

A total of 175 melon accessions were analyzed in two independent studies at West Virginia State University (WVSU_USA, 97 accessions [19]) and at Instituto de Biología Molecular y Celular de Plantas (IBMCP, 78 accessions this report,. Additional file 1: Table S1).

All the melon accessions were classified previously into seven groups based on their origin and/or passport information: African (7 accessions), cantaloupe (51 accessions), conomon (14 accessions), dudaim (4 accessions), Indian (37 accessions), inodorus-related (48 accessions; including cultivars from the *inodorus*, *ameri* and *flexuosus* botanical groups), and 14 accessions without a clear category that were classified as “ungrouped” (Additional file 1: Table S1). Accessions were further classified into e two subspecies subps. *Melo* and subps. *Agrestis* according to their horticulture group: ameri, cantalupensis, dudaim, flexuosus, inodurus, reticulatus, within subps. *Melo* and acidulous, arya, chito, conomon, kachri, tibish, wild-India and wild-Africa.

The accessions that belonged to India included: 15 snapmelons and 7 wild accessions from North India (IC, WM, [13, 16], respectively); 3 accessions from North East India (SM, [14]), 5 accessions from South India (AM, [15]) and, finally, 3 accessions from West India (ND-, [37]). These accessions were selected based on SSR genetic diversity analysis from a total of 155 accessions [13–16, 37] in order to focus on the IC- collection and to represent the genetic diversity of the other collections. Six additional accessions from different geographical regions were also included in the Indian group.

### SNP discovery by genotyping-by-sequencing

Genomic DNA from the IBMCP samples was isolated from young leaves with SpeedTools Plant DNA Extraction kit (Biotools, Spain). The GBS was performed by LGC Genomics GmbH (Germany) following the procedure reported by Elshire et al. [17]. Briefly, DNA was digested with the restriction enzyme *Ape*K I, barcoded libraries were prepared by accession and sequenced on an Illumina HiSeq 2000 platform.

SNPs were extracted using TASSEL-GBS Discovery/Production pipeline (https://bitbucket.org/tasseladmin/tassel-5-source/wiki/Tassel5GBSv2Pipeline). Only SNPs, not indels, with a minimum read depth of three were retained. Chromosomal assignment and position of SNPs on the physical map were deduced from the melon reference genome (version 3.5.1) at https://www.melonomics.net/ [38]. SNPs were designated by chromosome number and position. The GBS data from both analyses, WVSU and IBMCP, were merged based on the SNP genomic position. After merging, SNPs were filtered forMAF higher than 5% and present in at least 100 accessions (missing data lower than 57%). SNPs that showed a heterozygosity higher than 0.7 were also removed because they are likely are artifacts due sequencing to errors or duplicated sequences.

### Genetic structure and diversity

Before structure analysis, SNP matrix was pruned for linkage disequilibrium to avoid possible bias due to the variability associated to large haplotype blocks. Tag SNPs were defined with SNPAnalyzer 2.0 [39] The genetic structure of the collection was assessed by Multidimensional Scaling (MDS) with TASSEL 5.2 (Trait Analysis by aSSociation, Evolution and Linkage, www.maizegenetics.net) [40]and STRUCTURE [41] analysis. MDS was performed with TASSEL 5.2. The three first MDS components were plotted with CurlyWhirly (Information & Computational Sciences, The James Hutton Institute. https://ics.hutton.ac.uk/curlywhirly/). For STRUCTURE analysis, 20 independent runs for each K value ranging from 2 to 10 were performed with a burn-in length of 500,000 and 1 million iterations. The optimal subpopulation was calculated from the second order rate of change of likelihood (ΔK method) [42].

The genetic diversity was investigated with Arlequin ver. 3.1 [43]. Accessions were grouped according to subspecies (*C. melo* subsp. *agrestis* or subsp. *melo*) and the groups were defined after MDS analysis. Distribution of genetic variability among and within groups was calculated by Analysis Molecular of Variance (AMOVA) with two classification levels: subspecies and groups and also with only the MDS grouping classification. Expected (H_e_), observed (H_o_) heterozygosity and MAF were calculated for each group, as well as the Wright’s F statistic [44] F_ct_ (differentiation among subspecies) F_st_ (genetic differentiation among groups), and F_is_ (inbreeding coefficient). The ratio between high and low polymorphic SNPs was obtained by dividing the number of SNPs with MAF > 0.4 divided by the number of SNPS with MAF < 0.05 (MAF0.04/MAF0.05) for the whole collection and each group.

### Linkage disequilibrium (LD) and haplotype analysis

LD parameter r^2^ was estimated with TASSEL 5.2 for each SNP pair. LD decay was drawn as a smooth line of r^2^ against physical distance, fitted using a second-degree locally weighted scatterplot-smoothing LOESS implemented in an excel plugin [45], https://peltiertech.com/loess-smoothing-in-excel/). The r^2^ statistical threshold was established as the 95th percentile of the r^2^ distribution for unlinked SNPs. LD was analyzed for different subdata sets: whole collection, only SNPs in euchromatin, only SNPs in heterocromatin (according to [32, 38]), inodorus-related and cantaloupe groups.

Haplotype blocks were defined with SNPAnalyzer 2.0 [39]. Haplotype frequency within cultivar groups was calculated by custom scripts and visual inspection.

## Additional files

Additional file 1: **Table S1.** Details of the accessions analysed in the current study. Group, subspecies and type are according passport data. MDS group is according MDS analysis (nd, ungruped). The assay indicates the origin of the data: WVSU (Nimmakayala et al. 2016), IBMCP (current work). (XLSX 20 kb)

Additional file 2: **Table S2.** Single Nucleotide Polymorphsim genotypes after merging WVSU (Nimmakayala et al. 2016) and IBMCP (current work) data. (XLSX 5578 kb)

Additional file 3: **Figure S1.** MDS analysis including the whole germplasm collection. The origin of the GBS data is indicated by the dot color: blue for WVSU [19] and red for IBMCP. (PPTX 102 kb)

Additional file 4: **Figure S2.** STRUCTURE results for K = 2 to 5. The five groups defined by Multi Dimensional Scaling (MDS) analysis are indicated at the right with their respective colors. K = 5 showed the Delta K peak, defining five populations. Wild accessions are highlighted with a star with the color of their STRUCTURE populations. (PPTX 480 kb)

Additional file 5: **Figure S3.** Expected (H_e_) heterozygosity for SNPs across the melon genome in the groups Inodorus-related, Cantaloupe and Indian. (PPTX 765 kb)

Additional file 6: **Figure S4.** Histograms of the distribution of SNPs with different Minor Allele Frequencies (MAF) among Multidimensional scaling groups and the whole collection (all). (PPTX 83 kb)

Additional file 7: **Table S3.** Fst values for SNP loci for Inodurus-related-cantaloupe and Indian pairwise group comparisons (XLSX 283 kb)

Additional file 8: **Figure S5.** Linkage disequilibrium (r^2^) versus physical distance. (A) Whole germplasm analysing euchromatin and heterochromatin independently. (B) Inodorus-related and cantaloupe groups. Dashed lines indicate the false discovery rate at *p* < 0.05 based on the 95th percentile of the r^2^ distribution of unlinked SNPs. Curves were fitted by second-degree LOESS. (PPTX 158 kb)

Additional file 9: **Table S4.** Haplotypes blocks defined by SNPanalyzer 2.0 (XLSX 57 kb)

Additional file 10: **Figure S6.** Genomic location of LD blocks on the melon genome. Blocks fixed in *C. melo* ssp. *melo* (M) and cantaloupe group (C) are indicated. Centromeric regions in the chromosomes are marked in black. (PPTX 164 kb)

## Abbreviations

AMOVA: Analysis of molecular variance
GBS: Genotype-by-sequencing
GWAS: Genome-Wide Association Studies
H_e_: Expected heterozygosity
H_o_: Observed heterozygosity
LD: Linkage disequilibrium
LOESS: Local regression
MAF: Minor allele frequency
MDS: Multidimensional scaling
QTL: Quantitative trait locus
SNP: Single Nucleotide Polymorphism
SSR: Single Sequence Repeat
