# Supplementary figures and images for: Re-evaluation of the role of Indian germplasm as center of melon diversification based on genotyping-by-sequencing analysis
Source: BMC Genomics. 2019 Jun 3;20:448. doi: 10.1186/s12864-019-5784-0 (PMC6547464; doi:10.1186/s12864-019-5784-0)

## Slide 1
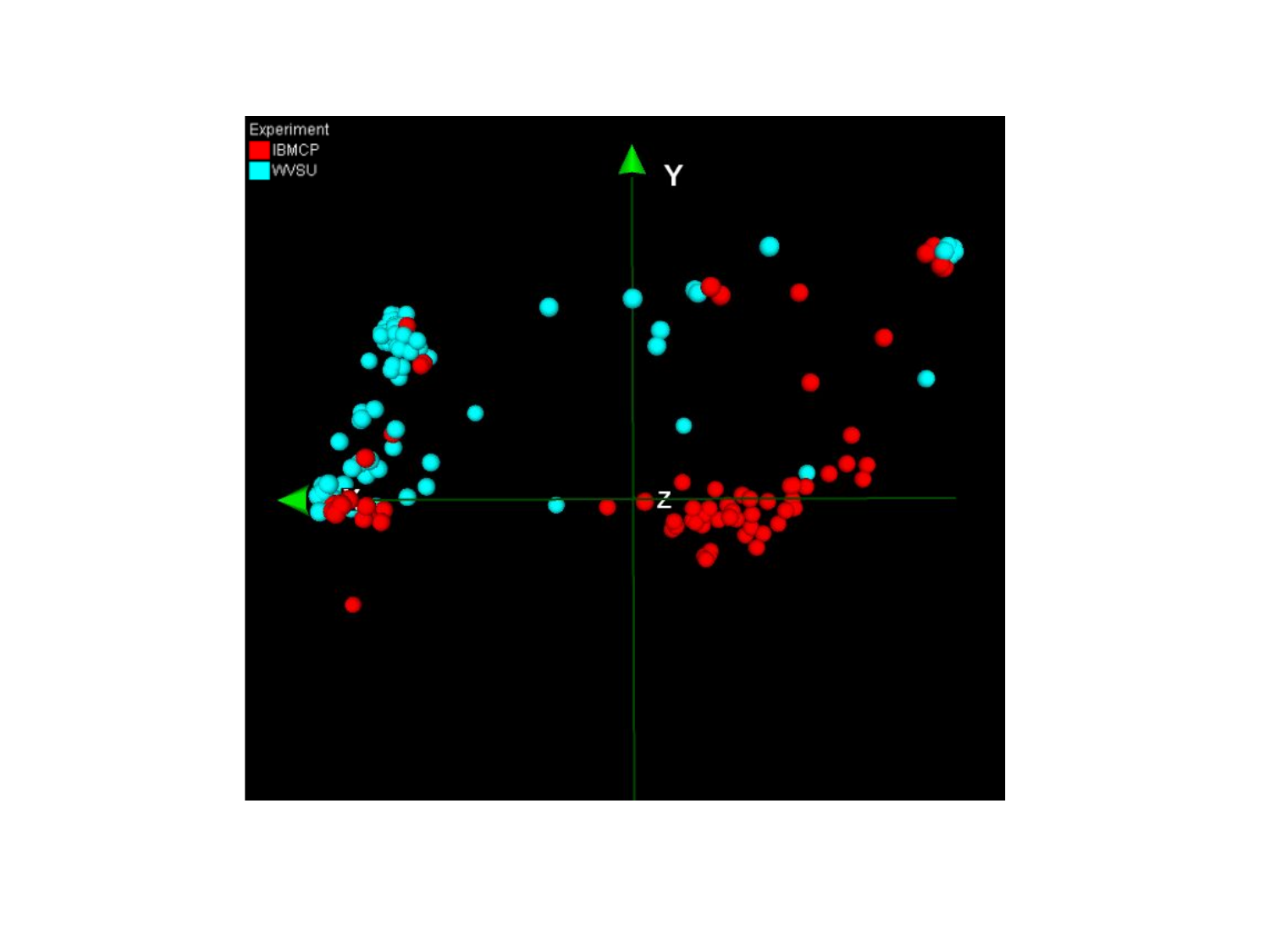

Supplement: Supplementary file 3 — Figure S1. MDS analysis including the whole germplasm collection. The origin of the GBS data is indicated by the dot color: blue for WVSU [19] and red for IBMCP. (PPTX 102 kb) [file 12864_2019_5784_MOESM3_ESM.pptx]

## Slide 1
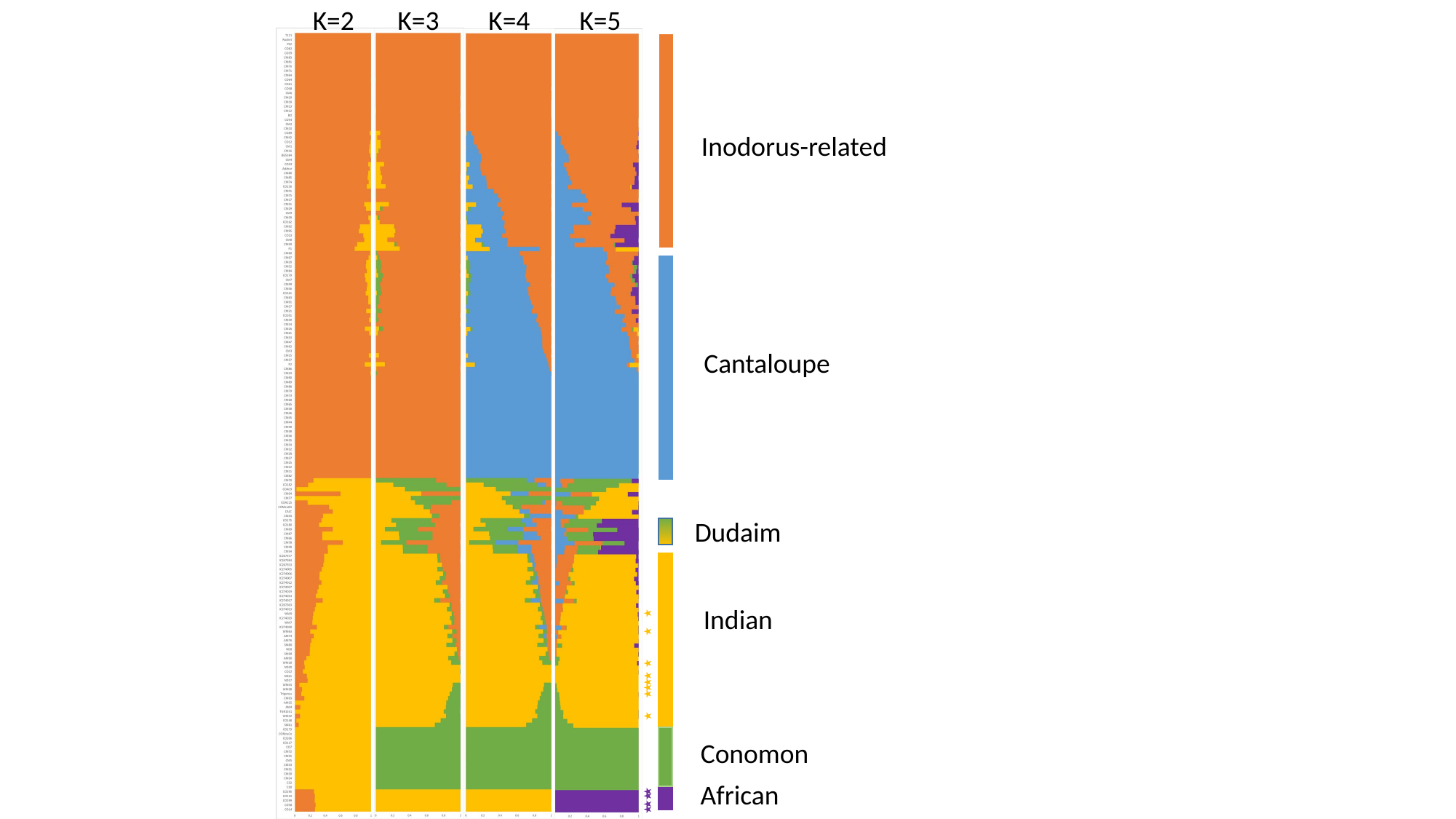

K=2 K=3 K=4 K=5
Inodorus-related
Cantaloupe
Dudaim
Indian
Conomon
African

Supplement: Supplementary file 4 — Figure S2. STRUCTURE results for K = 2 to 5. The five groups defined by Multi Dimensional Scaling (MDS) analysis are indicated at the right with their respective colors. K = 5 showed the Delta K peak, defining five populations. Wild accessions are highlighted with a star with the color of their STRUCTURE populations. (PPTX 480 kb) [file 12864_2019_5784_MOESM4_ESM.pptx]

## Slide 1
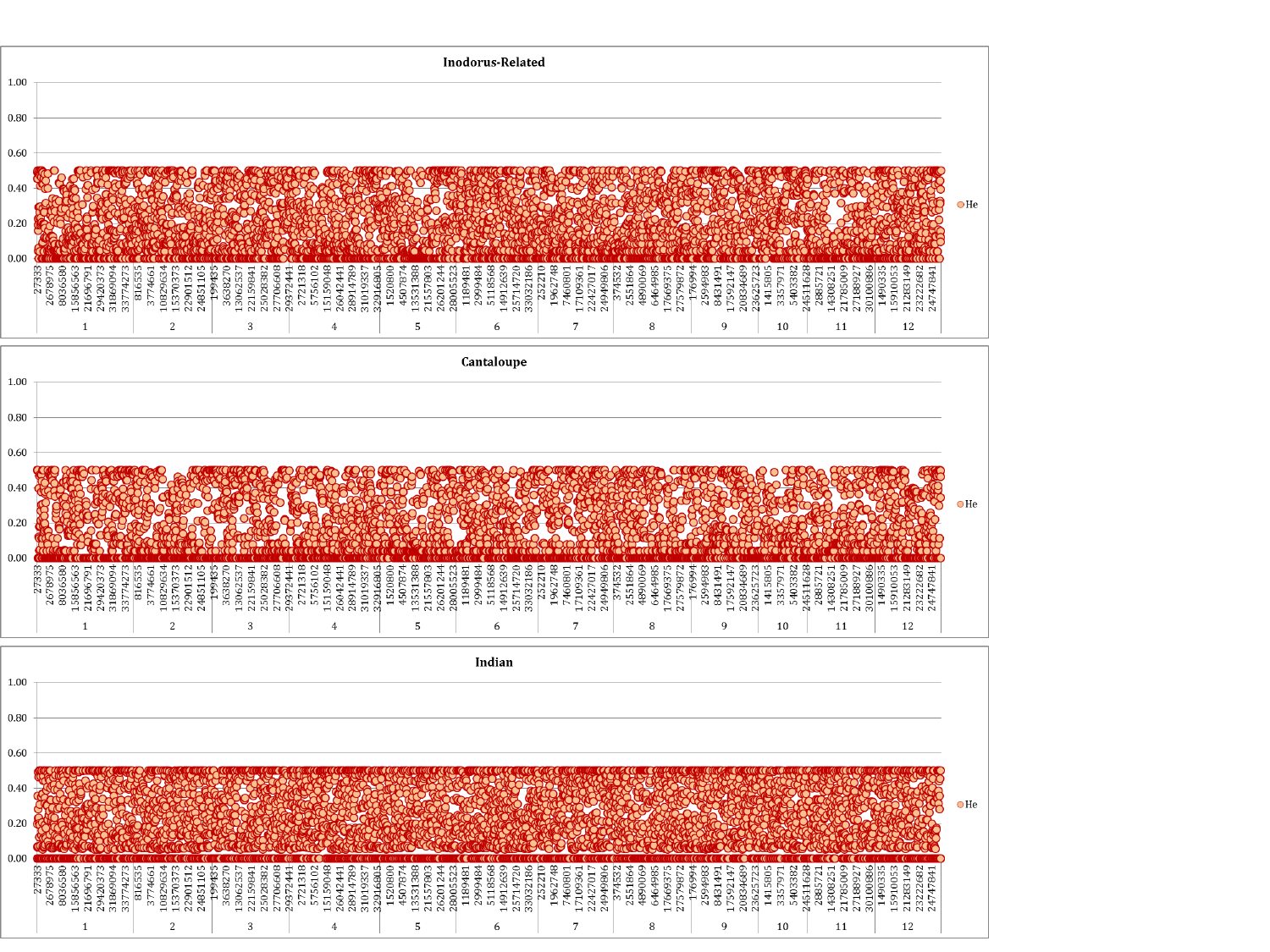

Supplement: Supplementary file 5 — Figure S3. Expected (He) heterozygosity for SNPs across the melon genome in the groups Inodorus-related, Cantaloupe and Indian. (PPTX 765 kb) [file 12864_2019_5784_MOESM5_ESM.pptx]

## Slide 1
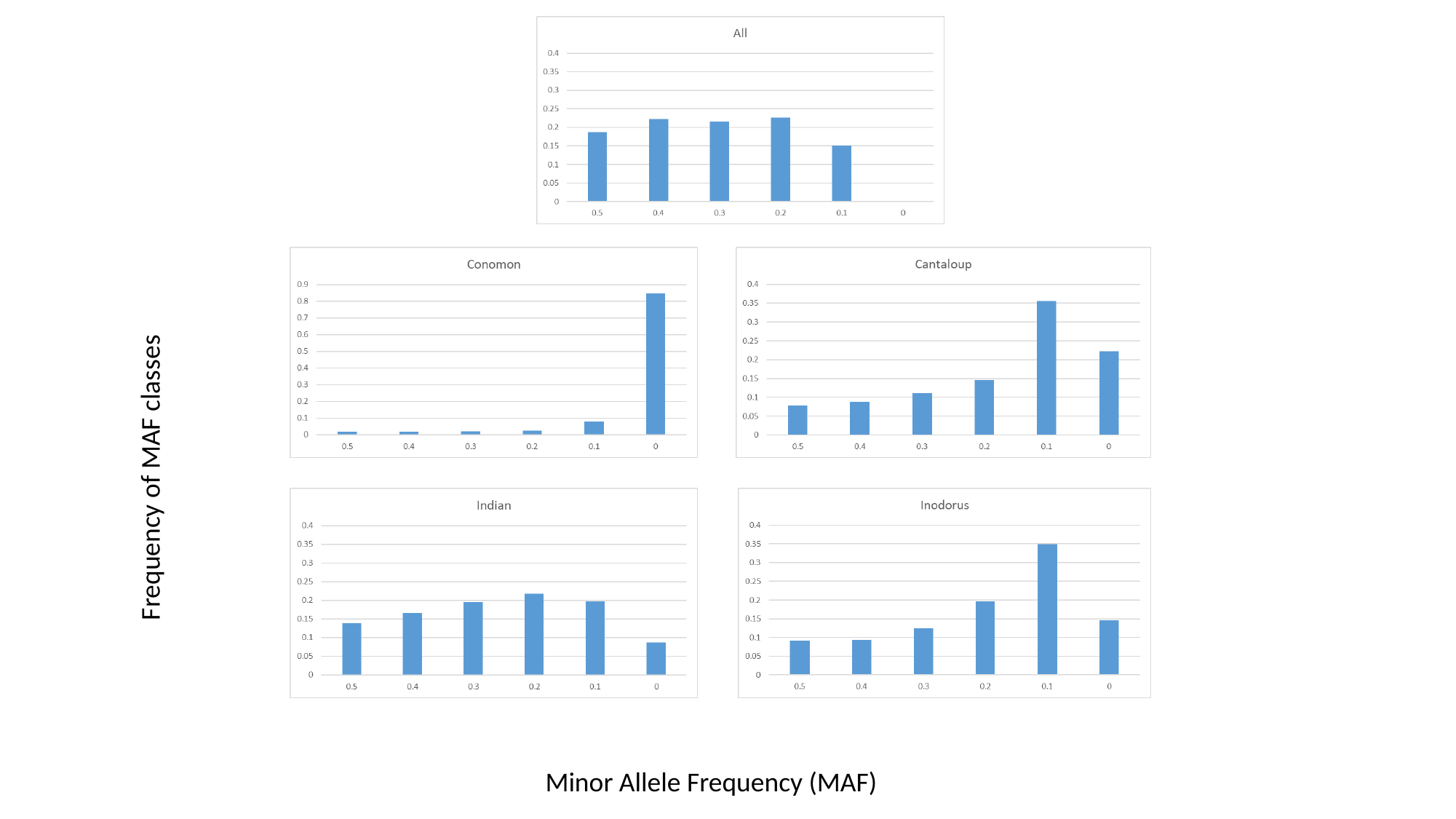

Frequency of MAF classes
Minor Allele Frequency (MAF)

Supplement: Supplementary file 6 — Figure S4. Histograms of the distribution of SNPs with different Minor Allele Frequencies (MAF) among Multidimensional scaling groups and the whole collection (all). (PPTX 83 kb) [file 12864_2019_5784_MOESM6_ESM.pptx]

## Slide 1
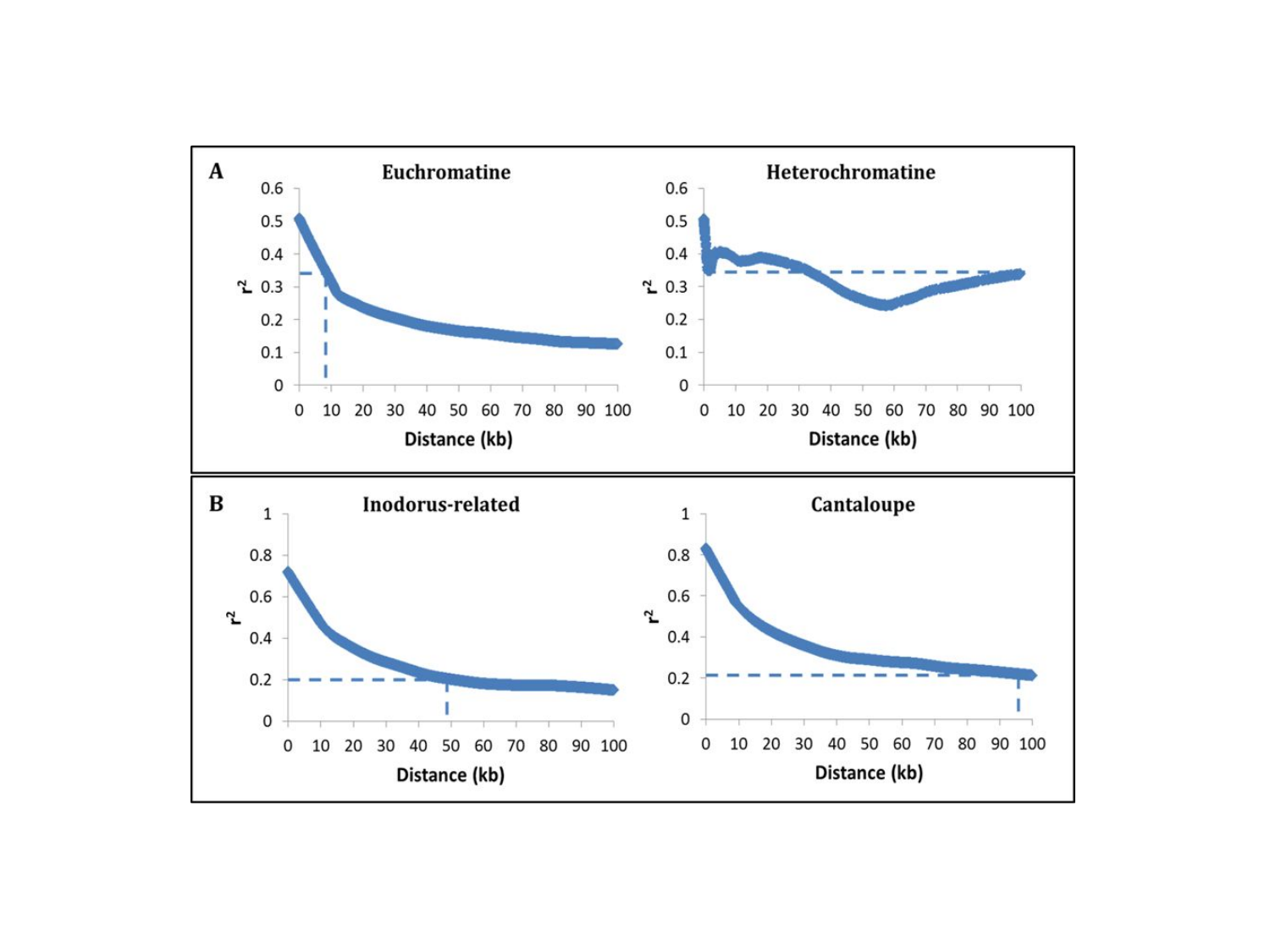

Supplement: Supplementary file 8 — Figure S5. Linkage disequilibrium (r2) versus physical distance. (A) Whole germplasm analysing euchromatin and heterochromatin independently. (B) Inodorus-related and cantaloupe groups. Dashed lines indicate the false discovery rate at p < 0.05 based on the 95th percentile of the r2 distribution of unlinked SNPs. Curves were fitted by second-degree LOESS. (PPTX 158 kb) [file 12864_2019_5784_MOESM8_ESM.pptx]

## Slide 1
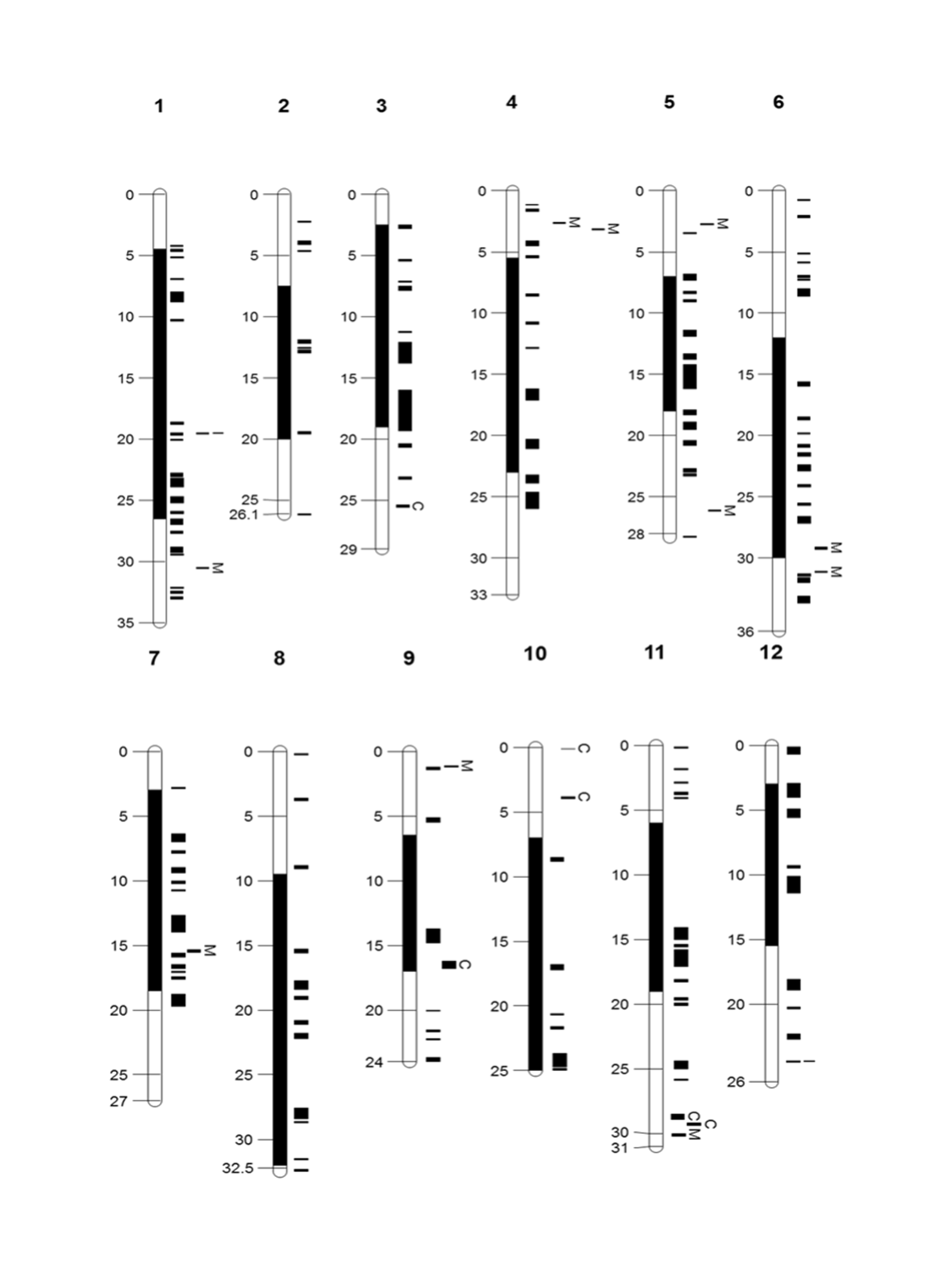

Supplement: Supplementary file 10 — Figure S6. Genomic location of LD blocks on the melon genome. Blocks fixed in C. melo ssp. melo (M) and cantaloupe group (C) are indicated. Centromeric regions in the chromosomes are marked in black. (PPTX 164 kb) [file 12864_2019_5784_MOESM10_ESM.pptx]
